# Characterizing factors influencing calibration and optical property determination in quantitative reflectance spectroscopy to improve standardization

**DOI:** 10.1117/1.JBO.27.7.074714

**Published:** 2022-04-07

**Authors:** Iris Schmidt, Wouter B. Nagengast, Dominic J. Robinson

**Affiliations:** a University Medical Center Groningen, Department of Gastroenterology and Hepatology, Groningen, The Netherlands; b Erasmus Medical Center, Center for Optical Diagnostics and Therapy, Department of Otorhinolaryngology and Head and Neck Surgery, Rotterdam, The Netherlands

**Keywords:** spectroscopy, calibration, standardization, optical properties, reflectance, fluorescence

## Abstract

**Significance:**

The combination of reflectance and fluorescence spectroscopy allows the determination of tissue optical properties and the calculation of the intrinsic fluorescence *in vivo*. These parameters can discriminate between tissues and may allow the discrimination of malignant from benign tissue. While this approach has significant clinical potential, the lack of standardization and quality assessment prevents the upscaling of research.

**Aim:**

Investigate which factors influence device calibration and tissue optical property determination. Improve system quality assessment and allow upscaling of the clinical research using multidiameter single fiber reflectance/singe fiber fluorescence spectroscopy.

**Approach:**

Two studies, one phantom based on uniform calibrations and skin measurements and a clinical study including clinical calibrations. The first validates the effect of factors under identical conditions and the effect of calibration quality on the optical property determination of skin. The second shows the effect of different system configurations and the performance of the system and probe over an extended period.

**Results:**

Phantom calibrations showed stability over a period of 20 weeks except for a 16-week-old intralipid phantom which showed a significant difference (at least p=0.0032) for all five probes evaluated. For clinical calibrations, only the fiber tree had a significant influence (probe 4: p<0.000001 and probe 5: p=0.00038) on the calibration quality. Interestingly, no degradation of probe performance was detected over a period of 21 months despite the exposure to stress during clinical measurements. Calibration quality affected $\mu_{s}^{\prime }$ and the power law scattering exponent, but the degree of the influence was different per fiber.

**Conclusions:**

Intralipid phantom quality and fiber tree performance are the main factors influencing the calibration quality. Probe and user performance did not show any effect, which makes the upscaling of research to multicenter trials easier. A high-quality assessment procedure should be implemented to track changes during clinical trials.

## 1 Introduction

Reflectance spectroscopy provides information about tissue-specific absorption and scattering optical properties. Differences in tissue optical properties can be used to differentiate between tissue types, and may, for example, allow the identification of malignant and benign tissue.^1^^–^^3^ Also, fluorescence spectroscopy has the potential to differentiate tissues based on their fluorescent properties but is often limited because it is strongly affected by the effects of background tissue optical properties that prevent quantitative fluorescence measurements. By combining both types of spectroscopies, the fluorescence signal can be corrected for the tissue’s optical properties, to yield the intrinsic fluorescence. This intrinsic fluorescence is an important parameter for clinical studies to compare the fluorescence intensity objectively within and between patients. It enables, for example, the determination of optimal tracer doses and the quantification of tumor-to-background ratios. The effect of different tracer doses can be evaluated in vivo without the distorting influence of the tissue, and by using targeted fluorescent tracers, tumor-to-background ratios can be used to make a distinction between benign and malignant or between inflamed and non-inflamed tissue.^4^^–^^8^ Although reflectance and fluorescence spectroscopy have great clinical potential, the lack of standardization and quality assessment of the system over time prevents upscaling of clinical research like multicenter trials using multiple devices.

In this study, we evaluated the sources affecting a custom-made multi-diameter single fiber reflectance/single fiber fluorescence (MDSFR/SFF) system. Quantitative MDSFR/SFF spectroscopy enables the extraction of tissue absorption and scattering properties (MDSFR) to the corrected measured fluorescence (SFF) for it.^9^^–^^11^ The system consists of an MDSFR probe, which is connected through a trifurcated fiber tree to a halogen lamp and two spectrometers for the reflectance measurements and via a quadfurcated fiber tree to a laser source and a third spectrometer for the fluorescence measurements. As the system must combine signals from two diameters and three individual spectrometers, the correct merging of these spectrometer channels is important for accurate determination of the optical properties and thus intrinsic fluorescence. Differences in spectral sensitivity and transmission efficiency are corrected by inserting the probe into an integrating sphere and applying uniform illumination using a halogen lamp. Additionally, two liquid phantoms, one is intralipid and another is water, account for the spectral illumination and transmission efficiencies and the spectrometer sensitivity for each effective probe diameter.

An MDSFR measurement consists of several co-localized single fiber reflectance (SFR) measurements of a turbid medium using two or more probe diameters. These SFR spectra are individually corrected for the effects of absorption, without prior knowledge of the scattering properties, resulting in the reflectance in the absence of absorption $R_{\mathrm{SF}}^{0}$.^12^ By acquiring SFR spectra with multiple probe diameters, an estimation can be made for the reduced scattering coefficient ($\mu_{s}^{\prime }$) and the phase function parameter (γ).^10^^,^^13^^,^^14^ The $\mu_{s}^{\prime }$ and γ can then be utilized in the individual SFR measurements to re-estimate the absorption coefficient ($\mu_{a}$). Ultimately, the absorption, reduced scattering coefficient, and phase function can be used to correct the fluorescence signal, resulting in the intrinsic fluorescence.^11^^,^^15^

Calibration of the device is performed before each procedure to account for the spectral illumination, transmission, and detection efficiencies of the device. However, also internal and external factors can influence the calibration and therefore the quality assessment over time. The effect of these factors on the calibration quality is unknown until now. A reduction in the calibration quality could influence the optical property and intrinsic fluorescence determination. Although not all influences on the device and its calibration can be fully prevented during a longer period of time, by determining the effect of these changes on the calibration and optical property determination it can be accounted for.

Small changes within the device, repairments of the device, and the MDSFR probe performance are the main internal factors influencing the calibration. The MDSFR probes are custom-made and manufactured as sterile probes. In our studies, sterility of the probes is often not necessary and therefore they are manually cleaned so they can be used multiple times. Also, these probes are used for both in vivo, endoscopically and surgically, and ex vivo purposes. During procedures, probes are exposed to bending, handling, and other risks which could induce degradation of the probe, possibly resulting in loss of signals. Endoscopically, the probe is exposed to even more stress. At insertion, the probe is bent but also at the tip of the endoscope it is manipulated. Additionally, the system can be set to different wavelengths by adjusting the fiber tree and corresponding filter sets. To change between wavelengths, the fiber tree has to be disconnected and switched which again increases the risk of degradation. The effect of these two internal factors on the calibration quality over a long period of time is unknown until now.

For the external factors, the intralipid phantom is the main component of variation combined with the number of users using the device. Often no standardized liquid phantoms can be purchased, and custom-made phantoms are used that can vary over time.^16^^,^^17^

Previously, solid phantoms, both white and black spectralon and silicone phantoms were explored for the calibration of this system.^13^ Using the spectralon standards (Labsphere SRS-99 and SRS-02) a set distance between the probe and spectralon surface is necessary. Maintaining this distance for each calibration is difficult, especially in a clinical setting. Silicone phantoms do not have this problem, as the probe can have direct contact with the phantom. However, this creates its own problem, as the pressure of the fiber on the phantom is difficult to maintain equal. Additionally, the surface of the solid phantoms can collect dust, possibly influencing the calibration. Therefore, we chose to use a liquid intralipid phantom for the calibration of this system. By submerging the fiber into the phantom, we eliminate the sensitivity to contact conditions and probe to phantom distances.

The custom-made intralipid solution is made from Intralipid 20% (Fresenius Kabi) and NaCl solution. Preparation of the solution by multiple users has the potential to induce variations in the quality of calibrations. Finally, the calibration is performed by multiple trained users, which may induce further variation between calibrations. Sufficient knowledge of the effect of both internal and external parameters is necessary to enable accurate measurements and ultimately to optimally determine the optical properties and intrinsic fluorescence, particularly when upscaling and multicenter trials using multiple devices are considered. Also, in large clinical trials, multiple researchers are involved in performing both the measurements and calibration. Although users are trained, inter- and intra-user variability remain a potential problem. By the evaluation of the sources affecting the calibration procedure and to what extent, the standardization and quality assessment of the device can be improved.

To establish the internal and external factors influencing the calibration, we performed two types of analysis. One based on static calibrations followed by skin measurements (fingertip) and one based on clinically performed calibrations. The first validates the effect of different sources under equal circumstances and the effect of calibration quality on the optical property determination. The second shows the effect of different system configurations and the performance of the probe and fiber tree(s) over time. We aim to investigate which factors influence the calibrations and if this translates to the determination of optical properties. This could improve the quality assessment of the system and allow upscaling of the clinical research using MDSFR/SFF spectroscopy.

## 2 Methods

**Fig. 1:**
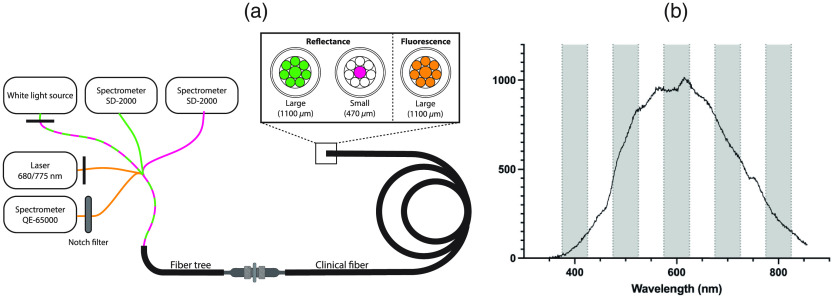
(a) Schematic overview of the MDSFR/SFF device. Each measurement consists of a reflection measurement with sequentially the small diameter (470  μm) and the large diameter (1100  μm), and a fluorescence measurement with the large diameter (470  μm). (b) An example of the raw signal from an intralipid phantom under white light illumination corrected for the dark background signal and corresponding water measurement.

The MDSFR/SFF device consists of a custom-made MDSFR probe (Light Guide Optics) designed to fit through the working channel of an endoscope. The probe contains eight optical fibers for both delivery and collection of the reflected light (Fig. 1). These eight fibers can be used to acquire reflectance spectra for two effective diameters, small diameter of 470  μm and a large diameter of 1100  μm. Each probe is polished at an angle of 15% to minimize the internal specular reflections from the probe tip. For each measurement, first, a reflection spectrum is acquired for each effective probe diameter (pink and green) followed by the acquisition of fluorescence spectra from the large effective diameter (orange). White light illumination is provided by a tungsten halogen light source (HL-2000-FHSA, Ocean Optics, Duiven, The Netherlands) directed through the first leg of the five-legged fiber tree into the MDSFR probe and into the tissue. The reflected light is collected by the same probe and directed via another leg of the five-legged fiber tree into one of the two spectrometers (SD-2000, Ocean Optics) for detection. Fluorescence excitation is achieved by guiding light from a laser source via a fourth leg of the fiber tree and a large effective diameter onto the tissue. The scattered fluorescence light is collected via the last leg of the fiber tree into a third spectrometer (QE-65000, Ocean Optics), where a notch filter blocks excitation light. Both light sources (halogen and laser) have computer-controlled shutters to regulate the illumination. For each of the studies described below, spectra were acquired using the same integration time and the same number of spectral averages.

In this study, we evaluated four clinically used MDSFR probes and one new, unused MDSFR probe. Table 1 shows the probe specifications and the number of procedures that were performed with the probe and for which application they were used, endoscopic procedures, surgical procedures (*in vivo*), or surgical specimen (*ex vivo*). Additionally, because of damage to the fiber tree during the clinical studies, two fiber trees have been used consecutively. The effect of the use of both on the calibration quality was also evaluated.

**Table 1:** Characteristics for the MDSFR probes.

Probe	Length (m)	Number of procedures	Period of usage	Application	Number of users
Probe 1	2.4	0	—	Unused	1
Probe 2	2.4	47	October 2019 to June 2021	Endoscopic	6
Probe 3	2.4	45	September 2019 to May 2021	Endoscopic	5
Probe 4	2.4	67	Sepember 2019 to May 2021	Surgical	3
Probe 5	1	>100	September 2019 to May 2021	*Ex vivo*	10

### 2.1 Extraction of Optical Properties

The extraction of optical properties from an MDSFR measurement was previously described.^18^

In short, for each SFR measurement, the tissue absorption coefficient $\mu_{a}$, is determined by a modified Beer–Lambert law relationship

$$R_{\mathrm{SF}}=R_{\mathrm{SF}}^{0}e^{-\mu_{a}L_{\mathrm{SFR}}}.$$ (1)

With the model for the effective SFR path length

$$\frac{L_{\mathrm{SFR}}}{d_{f}}=\frac{C_{\mathrm{PF}}p_{1}}{\left(\mu_{s}^{\prime }{)}^{p_{2}}[p_{3}+(\mu_{a}d_{f}{)}^{p_{3}}\right]},$$ (2)

and the model for $R_{\mathrm{SF}}^{0}$

$$R_{\mathrm{SF}}^{0}=\eta_{\lim}(1+p_{6}e^{-p_{4}\mu_{s}^{\prime }d_{f}})[\frac{(\mu_{s}^{\prime }d_{f}{)}^{p_{5}}}{p_{4}+(\mu_{s}^{\prime }d_{f}{)}^{p_{5}}}],$$ (3)

including a background scattering model. The collection efficiency at the diffusion limit is described by $\eta_{\lim}$. This is given at 2.7% for a probe numerical aperture of 0.22 in a medium of refractive index 1.38.^19^ The product of terms preceding the square brackets represents the single-fiber collection efficiency. The term within the square brackets describes the saturation relationship between $R_{\mathrm{SF}}^{0}$ and $\mu_{s}^{\prime }d_{f}$ that showed phase function independent behavior for high dimensionless scattering values. The fitted parameters $\left[C_{\mathrm{PF}},p_{1},p_{2},p_{3}\right]$ and $\left[p_{4},p_{5},p_{6}\right]$ were derived from previously performed Monte Carlo simulation.^13^^,^^20^ The values [0.944, 1.54, 0.18, 0.64] for $\left[C_{\mathrm{PF}},p_{1},p_{2},p_{3}\right]$ and the values [6.82, 0.969, 1.55] for $\left[p_{4},p_{5},p_{6}\right]$ were found to minimize the residual error between the SFR model and the simulations.

### 2.2 Calibration Method

First, the probe is inserted into an integrating sphere to measure the spectra for all spectrometers under uniform illumination. These spectra are used to correct the spectrometer channels for differences in spectral sensitivity and transmission. Next, the spectra are corrected for the spectrometer sensitivity for each effective probe diameter. This step is achieved by sequentially submerging the probe into two liquid phantoms, a 2% Intralipid solution, and a dark container with water.^21^

The spectra from the intralipid phantom ($I_{\mathrm{cal}}^{\mathrm{eff}}$) are compared with the absolute reflectance for this phantom ($R_{\mathrm{cal}}^{\mathrm{sim}}$) simulated by a Monte Carlo model for each probe diameter. The water phantom spectrum ($I_{\mathrm{water}}^{\mathrm{eff}}$) emerges from back reflections within the system and is subtracted from every measurement. The resulting measurement is calibrated into absolute reflectance ($R_{\mathrm{SF}}$) for each probe diameter independently, where

$$R_{\mathrm{SF}}=R_{\mathrm{cal}}^{\mathrm{sim}}\frac{I_{\mathrm{meas}}^{e\mathrm{ff}}-I_{\mathrm{water}}^{\mathrm{eff}}}{I_{\mathrm{cal}}^{\mathrm{eff}}-I_{\mathrm{water}}^{\mathrm{eff}}}.$$ (4)

Equation (4) is used to calibrate the reflectance spectra from each effective probe diameter independently.

### 2.3 Tripod

A tripod was designed and custom-made by the Research & Support Facility of the University Medical Center Groningen. This tripod holds the probe for calibration measurements, reducing the movement during the measurement. Without the tripod, the probe is held by the user.

### 2.4 Phantom Study

We evaluated the five MDSFR probes and 10 intralipid phantoms with different shelf lives under identical conditions. All phantoms were prepared to calibrate the system and perform measurements for several clinical studies and, therefore, were made by multiple users. The intralipid phantom consisted of 2.64 mL Intralipid 20% (Fresenius Kabi) with 37.36 mL 0.9% NaCl, resulting in a total of 40 mL of 2% Intralipid. This phantom was stored in a clear plastic, cylindrical container with a volume of 40 mL at 4°C. As these intralipid phantoms were prepared and saved over a period of 20 weeks, different batches of Intralipid 20% were used. In total, 10 Intralipid phantoms were saved over time, ranging thus from same-day preparation to 20 weeks after preparation. The solutions were stored at 4°C between measurements, which were performed at room temperature. For each probe, five calibrations were performed for each intralipid phantom. Each calibration was followed by a set of five skin measurements where the probe was placed on the fingertip of a single user and held stationary between measurements. These skin measurements mainly consist of scattering signatures that are relatively similar between people. Therefore, reflectance spectra of these skin measurements are already clinically used as an extra verification of the calibration quality.

### 2.5 Clinical Study

We investigated the calibrations performed for several clinical pilot studies. These were performed by 11 users for four probes between October 2019 and June 2021. Calibration was performed before each new (clinical) procedure up to 24 h, without disconnecting the fiber tree or MDSFR probe, before the clinical measurement in a dark environment.

### 2.6 Data Reduction and Statistical Analysis

#### 2.6.1 Calibration quality

A custom-made MATLAB (release 2018a, The Mathworks, Natick, Massachusetts, United States) script was used for the analysis of the calibration data for both the phantom and clinical study. The calibration quality was calculated as the mean signal-to-noise ratio (SNR) (intralipid signal/std intralipid), which describes changes of the signal over time. These changes can be due to phantom degradation, probe degradation, and fiber tree deviations. A 50 nm bandwidth centered on 700 nm from the Intralipid reflectance spectrum was chosen for this calculation. Although the SNR showed the highest signal around 600 nm, this also included the highest variation within the spectrum (Fig. 1). The wavelength of 700 nm was the second-highest and showed a linear decrease in the intensity of signal over this wavelength band.

#### 2.6.2 Optical property determination

The optical properties were estimated as described in Sec. 2.1. Two previously performed studies showed very low blood volume fraction (BVF) in superficial skin measurements.^3^^,^^22^ When the BVF is low, the measurements are dominated by the scattering properties. Therefore, in this study, we only show the results of two scattering properties; $\mu_{s}^{\prime }$ and the power law exponent. Three exclusion criteria were applied to reflectance spectra used to determine the optical properties of skin: (1) an absolute residual >25, (2) a blood volume fraction >40%, and (3) a mean saturation (${\mathrm{StO}}_{2}$) confidence interval of more than three standard deviations from the mean. If data agreed with one of these criteria, the measurement was excluded from the analysis. The weighted means of the tissue optical property parameters were calculated by averaging repeated measurements per calibration file by the individual confidence intervals. This resulted in five mean values per intralipid phantom.

#### 2.6.3 Statistical analysis

Differences between the two groups were analyzed using an unpaired t-test (normally distributed) or a Mann–Whitney test (non-normally distributed data). Differences between multiple groups were analyzed using ANOVA. Simple linear regression was performed to determine the signal over time. A p-value below 0.05 (two-sided) was considered statistically significant. Statistical analysis was performed using GraphPad Prism (version 9.2, GraphPad Software, La Jolla, California, United States).

## 3 Results

### 3.1 Data Overview

**Fig. 2:**
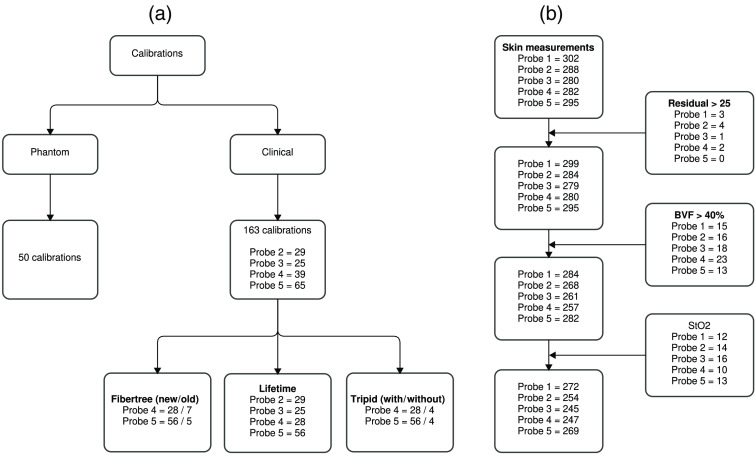
Flowchart of the included (a) calibrations and (b) skin measurements used for analysis.

In total, 250 calibrations were performed for the phantom study and 163 for the clinical study [Fig. 2(a)]. The former included five calibrations per intralipid phantom, resulting in 50 calibrations for each MDSFR probe. All measurements were measured sequentially for each probe, starting with the newest intralipid phantom first. The clinical calibrations were gathered from the clinically performed calibration files performed at the time of measurement. Calibrations were excluded where the probe type or user who performed the calibration was unknown. Ultimately the calibrations were divided into three groups: the first evaluated the two fiber trees, the second evaluated the added value of a tripod during the calibration, and the third evaluated the probe and fiber tree performance over a period of 21 months. Calibrations from the second and third groups only include measurements with the same type of fiber tree configuration. All measurements were performed with the use of a tripod except if stated otherwise.

A total of 1447 skin measurements were performed for all probes [Fig. 2(b)]. The number of skin measurements per calibration varied between five and eight measurements. Extra measurements were taken if there was uncertainty about the quality of the measurement, due, for example, to probe movement. The three exclusion criteria described above resulted in a removal of 1.4%, 8.2%, and 6.1% of the measurements for the residual, BVF, and ${\mathrm{StO}}_{2}$, respectively. The remaining measurements were used to calculate the optical properties of skin and evaluate the performance of the fiber tree and MDSFR probes.

### 3.2 Phantom Study

**Fig. 3:**
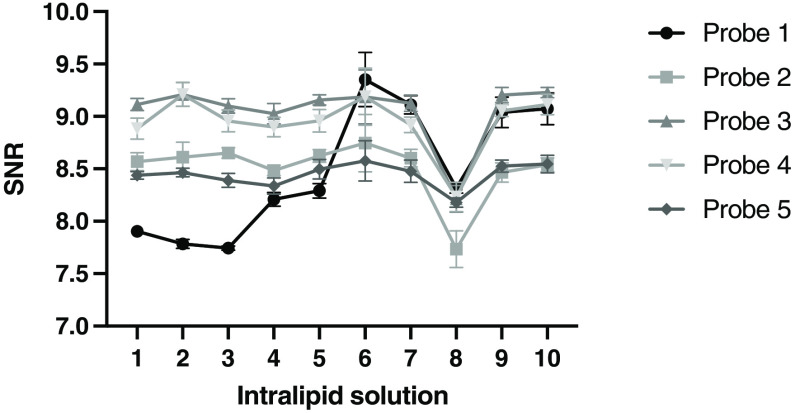
Intra- and inter-phantom variability for five probes. Probe 1 showed a significant difference for all intralipid phantoms, except for phantom 2, compared to the first intralipid phantom. For solution 8, probe one shows a p-value of 0.0010. Probes 2 to 5 show only a significant difference for solution 8, p=0.0008, p=0.0002, p=0.0005, and p=0.0032, respectively.

To determine the variability between the intralipid phantoms with different preparation dates, all probes were compared intra- and inter-phantom for the measured SNR values (Fig. 3). Overall, the intra-phantom variability was below 4% and the inter-phantom variability below 9% for all probes. For all five probes, the highest intra-phantom variability (2.2% to 3.1%) was found in phantom 6. Only probes 1 and 2 showed the lowest values for the variability in phantom 1, as expected. For probes 2 to 4, the lowest values were measured for phantom 4, 10, and 9, respectively. Additionally, phantom 1 was set as a reference phantom for the inter-phantom variability as it should be the most optimal one. Only phantom 8 showed a statistically lower SNR between the first and eighth intralipid phantoms for all probes (Fig. 3). Interestingly, all intralipid phantoms measured with probe 1 deviated significantly from the first intralipid solution, except for the second phantom.

**Fig. 4:**
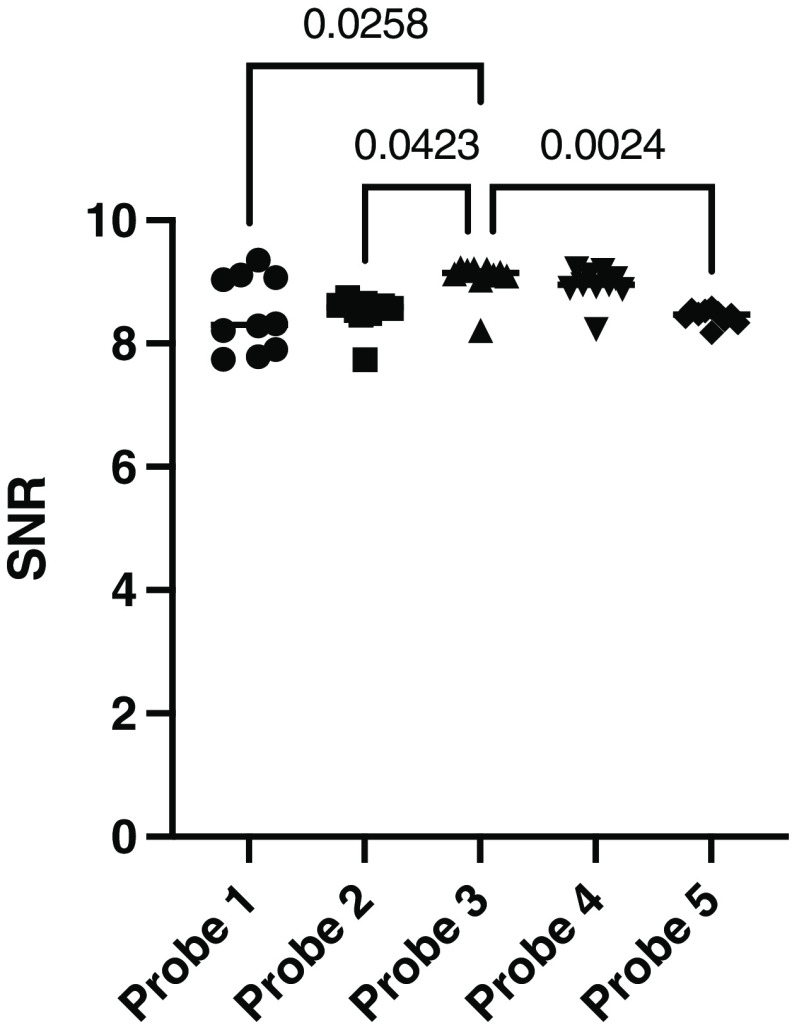
The SNR values for all 10 intralipid phantoms per probe. Median values are plotted. p-values were calculated with a Kruskal–Walis test.

Figure 4 shows the SNR values for all ten intralipid phantoms per probe. Probe 3 shows a higher median SNR compared to all others, which was statistically different for probes 1, 2, and 5 (p=0.0258,p=0.0425, and p=0.0024, respectively). The high inter-phantom variability within probe 1 is also clearly visible here.

Intralipid solutions remained stable until 20 weeks after preparation. Only for the 16-week-old phantom, the calibration quality was reduced. Therefore, the influence of the intralipid phantom is minimal as long as it is kept free from contamination. Moreover, a difference in calibration quality per probe was measured, which was only significant for probe 3, compared to probes 1, 2, and 5. This difference appears to be a certain factor per probe, which could possibly be corrected by establishing the initial performance of the probe.

### 3.3 Clinical Study

**Fig. 5:**
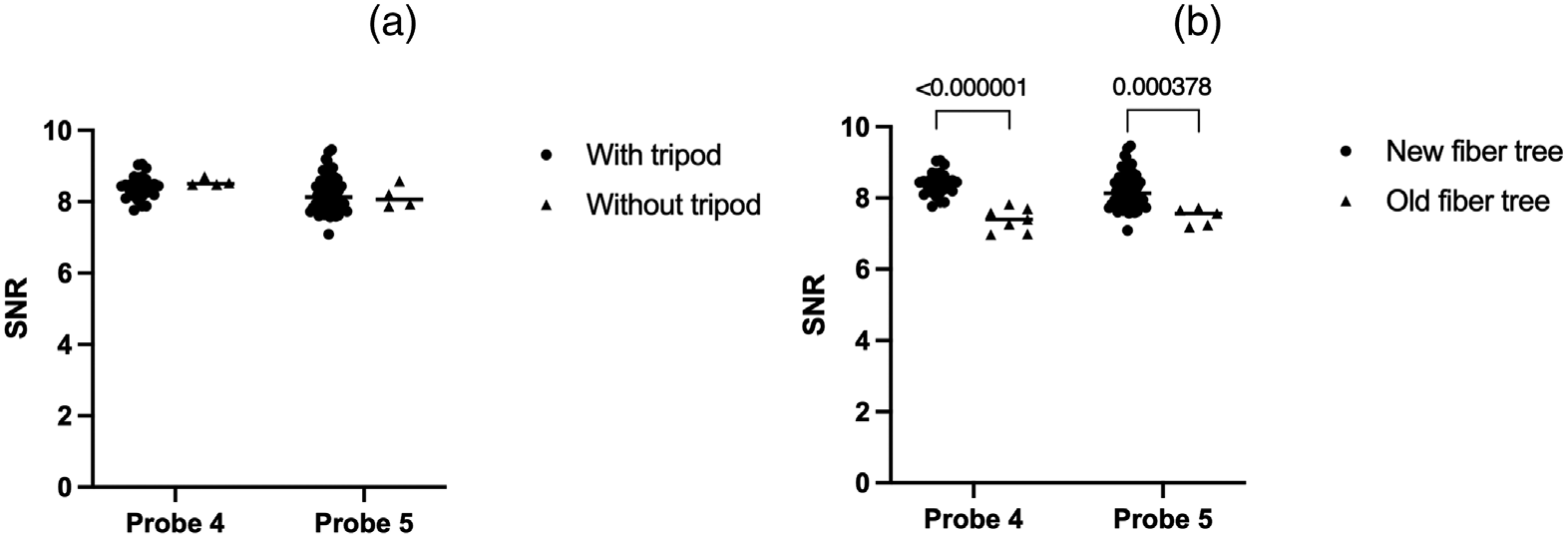
The effect of (a) the tripod and (b) fiber tree for two probes. p-values were calculated using a multiple Mann–Whitney test.

For the clinical calibration, the influence of the type of fiber tree with or without a tripod and the changes of the probe over the evaluated time period was assessed using the calibration quality. The tripod and fiber tree could only be evaluated with two probes as the other probes were not used in both situations. However, both the tripod and fiber tree groups had a limited number of measurements in the “without tripod” and “old fiber tree” groups, respectively (Fig. 5). The tripod was utilized during calibration to stabilize the probe during the phantom measurements as the movement of the probe during these measurements could decrease the calibration quality. Nevertheless, the usage of a tripod did not show an effect on the measurement stability and therefore the calibration quality (p=0.13 and p=0.92). On the other hand, the fiber tree did show a statistical difference between the old and new fiber trees for both probes (p<0.000001 for probe 4 and p=0.00038 for probe 5).

**Fig. 6:**
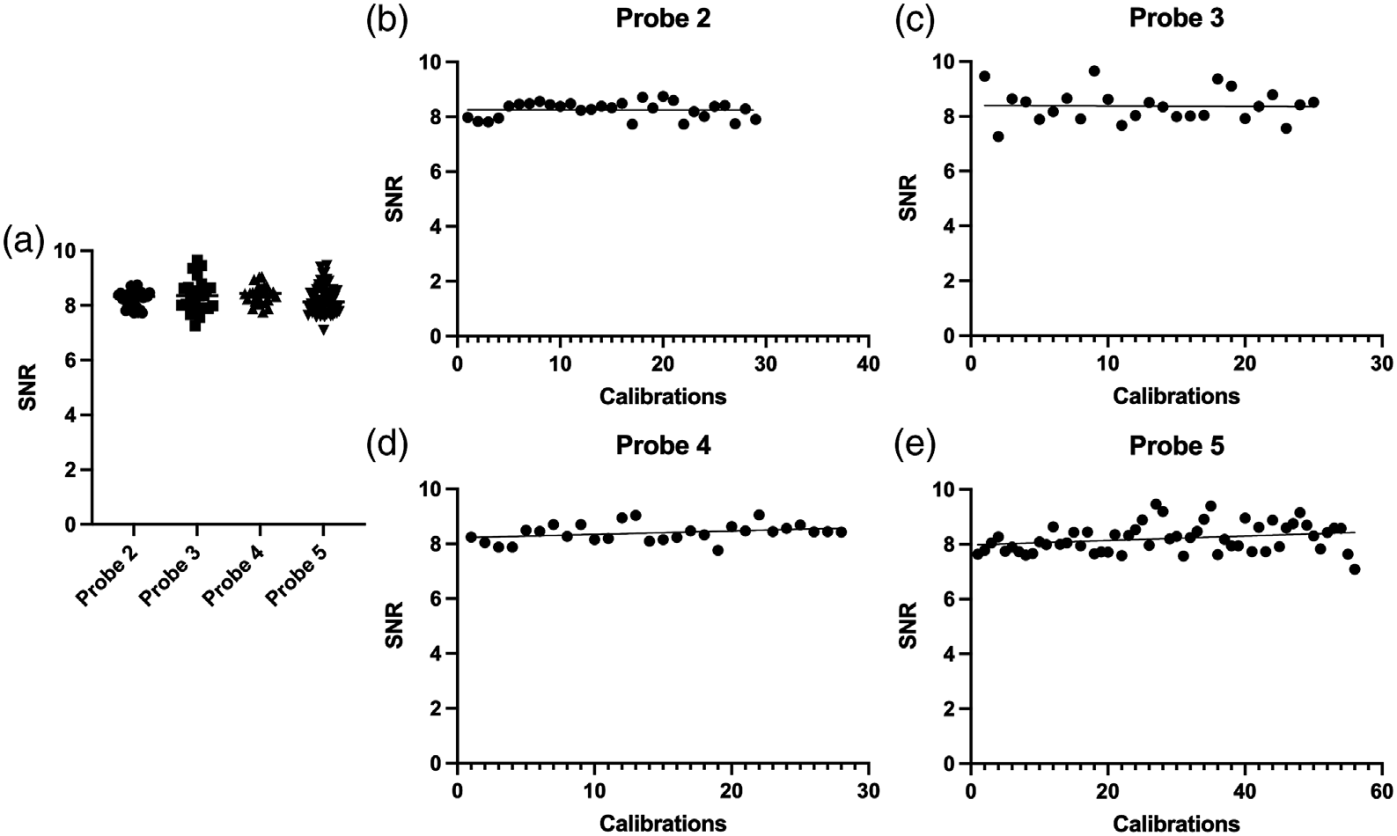
(a) All clinical calibrations together per probe and (b)–(e) the measured SNR per calibration over a time period including the linear regression line.

Finally, the probes were evaluated over a specific period of time. Collating the calibrations performed per probe did not demonstrate a difference in the mean SNR [Fig. 6(a)]. Also, the variability between calibrations was below 8% for all probes. Probe 3 showed the highest variability (7.0%) and probe 2 the lowest (3.7%). Using linear regression, the change in SNR over time was evaluated for each probe individually [Figs. 6(b)–6(e)]. None of the probes showed a significant difference between the SNR over the evaluated period.

Evaluating the performance of the system over a period of 21 months showed that only the performance of the fiber tree influenced the calibration performance. The use of the tripod did not improve user calibration quality. Interestingly, although probes are prone to stress during the clinical measurements, this does not seem to influence their performance.

### 3.4 Optical Properties

After the removal of the spectra that matched the exclusion criteria mentioned above, the remaining skin measurements were assessed. It is important to note, all skin measurements were performed by one user and the probe was not moved between measurements for the same calibration. For each set of five skin measurements per calibration, the scattering and absorption parameters were evaluated by calculating the weighted mean. As skin measurements are dominated by the scattering parameters, only the reduced scattering coefficient at 800 nm and the power law exponent were used for further analysis. For these two parameters, the weighted mean was calculated per calibration, resulting in five mean values per intralipid phantom.

**Fig. 7:**
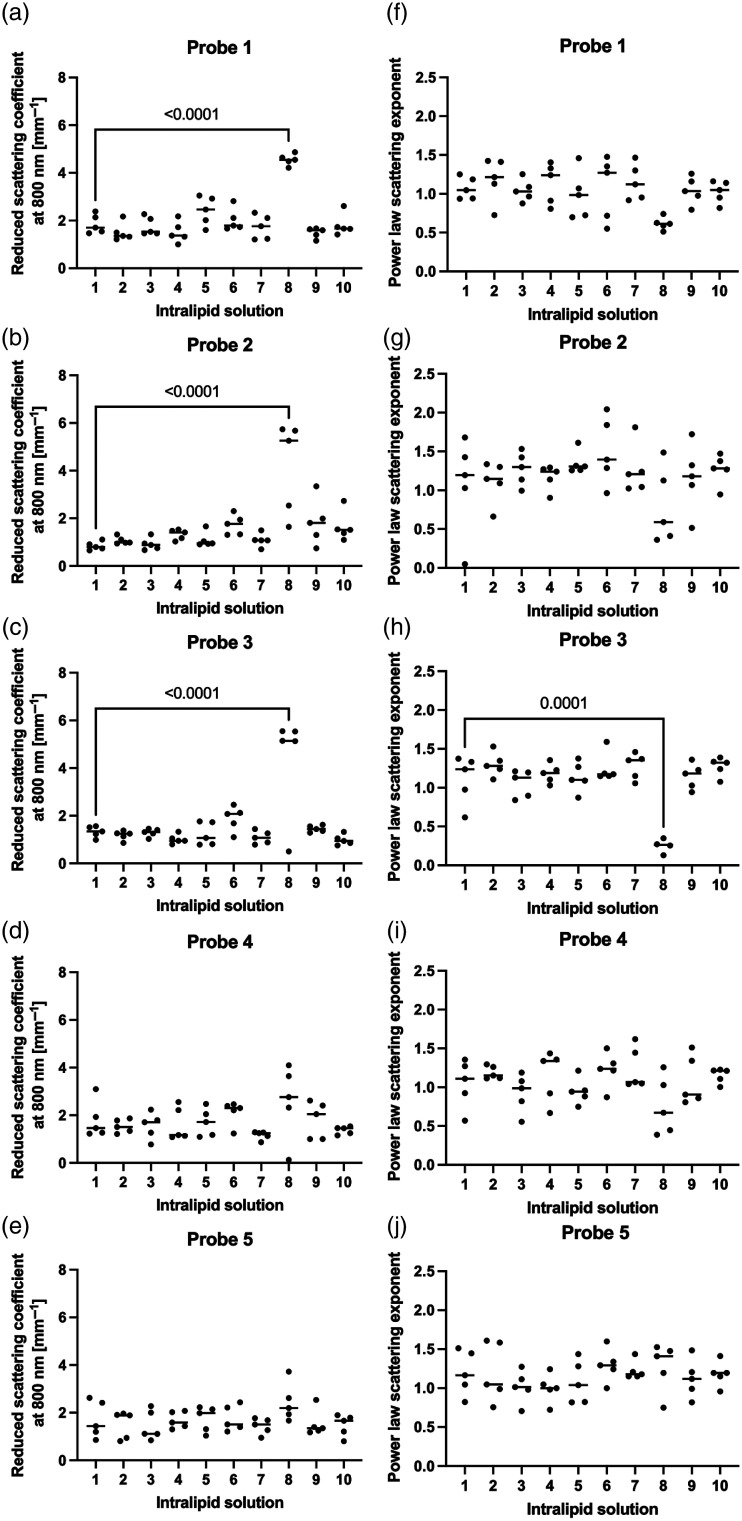
The weighted means for the (a)–(e) reduced scattering coefficient and (f)-(j) power law exponent.

Figures 7(a)–7(e) show the weighted mean for the reduced scattering coefficient per intralipid phantom per probe. For the inter-phantom calculations, which were only compared to the first phantom, phantom eight showed a higher SNR for all probes. This was statistically different (p<0.0001) for probes 1 to 3. For the power law exponent [Figs. 7(f)–7(j)], the SNR of phantom 8 is lower compared to the other phantoms for probes 1 to 4, and only statistically different for probe 3 (p<0.0001). Interestingly, probe 5 showed a higher SNR for phantom eight compared to the other phantoms. When correcting the weighted means for the probe, no differences were found for either parameter.

Table 2 shows the intra-phantom variability mean and standard deviation for both parameters per probe. The intra-phantom variability is higher for these parameters compared to the values for the calibration. Probes 1 to 3 show comparable values for both the reduced scattering coefficient and the power law exponent, where probe 3 showed the lowest variability for both parameters.

**Table 2:** Extracted means and standard deviations for the intra-phantom variability for the reduced scattering coefficient and power law exponent.

	Probe 1		Probe 2		Probe 3		Probe 4		Probe 5	
Parameter	Mean	SD	Mean	SD	Mean	SD	Mean	SD	Mean	SD
$\mu_{s}^{\prime }$	22.1	7.3	29.6	12.6	19.4	9.8	32.7	15.2	33.2	7.8
Power law exponent	20.8	8.6	29.3	18.0	17.2	8.4	23.8	12.2	20.8	6.40

### 3.5 Users

**Fig. 8:**
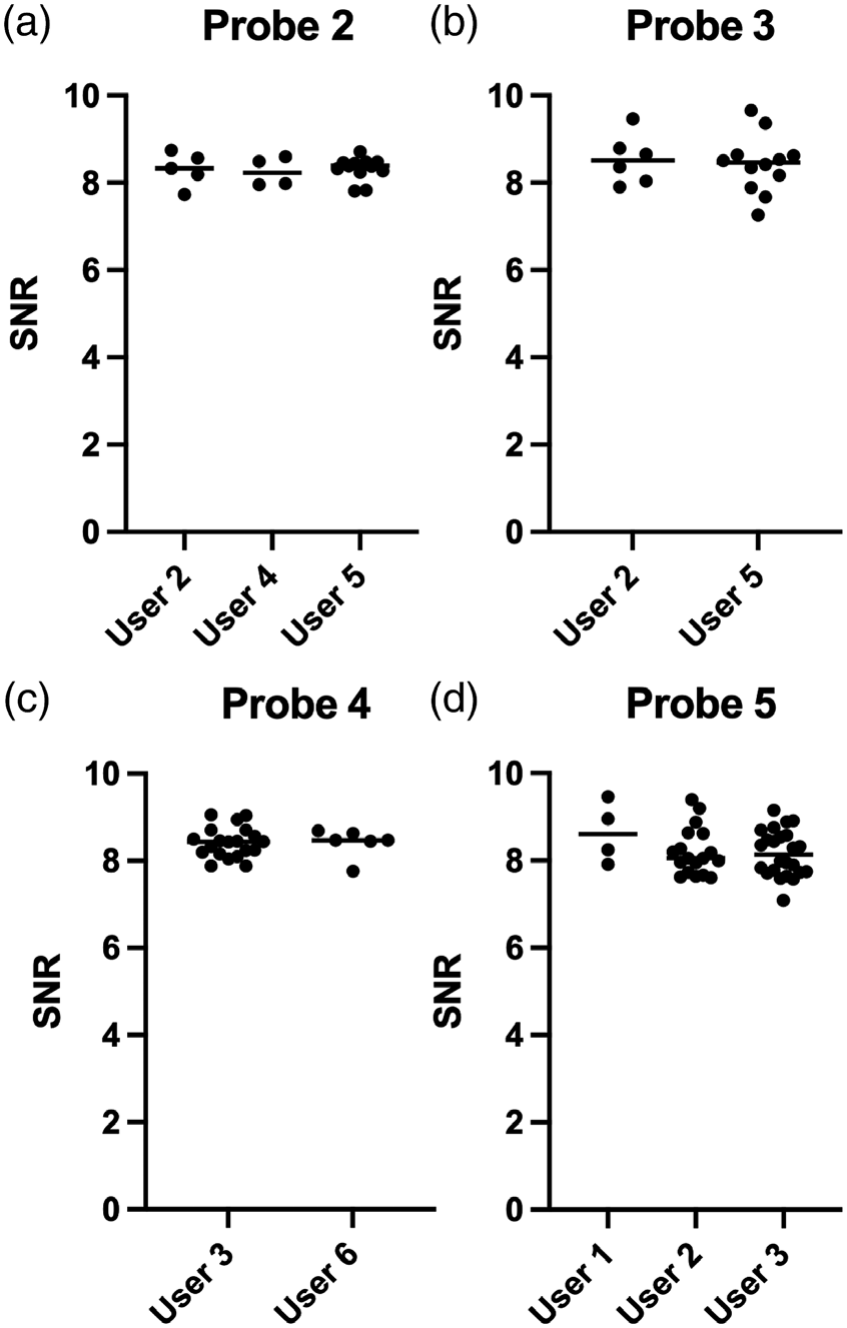
The calibration quality between users for all four clinical probes: (a) probe 2, (b) probe 3, (c) probe 4, and (d) probe 5.

To determine the variability between users, the spread in calibration quality was evaluated for six individual users. Each user should have performed a minimum of four calibrations for the evaluated probe. None of the probes showed statistically significant a user-dependent difference for the calibration quality (Fig. 8).

## 4 Discussion

Standardization of the calibration procedures and system evaluation over time are important factors to provide upscaling of clinical research. In this study, we investigated which internal and external factors affected the calibration quality (SNR) and the optical property determination of skin during MDSFR/SFF spectroscopy. Our results show that the intralipid phantom sterility and fiber tree performance are the main factors affecting the calibration quality. The intra- and inter-phantom variability for the calibration quality was stable and below 10% for both the phantom and clinical study. A contaminated intralipid phantom can affect the calibration quality as shown by phantom 8 in the present study. Although the fiber tree was expected to be a passive component of the system, having no influence on performance, we have shown that it can have an impact on the calibration quality. The probe, on the other hand, did not show any degradation of performance over a period of 21 months despite this component being much more stressed during clinical measurements. Finally, our study of different users demonstrated that a stable calibration can be achieved without the need for a tripod holding the probe during the calibration.

The intralipid phantom study present here showed that careful handling and storage of the intralipid phantom is important. Since we evaluated the calibration quality under uniform circumstances, other influences such as measurement environment, users and probe performance were limited. Therefore, differences measured in the intra- and inter-phantom measurements could only be the result of the intralipid phantom itself. This suggests that the deviations found in phantom 6 (intra-phantom) and phantom 8 (inter-phantom) are the results of contamination of the phantom itself. This is supported by the fact that similar deviations are found for all five probes. However, if careful preparation and storage of the phantom are taken into account, it shows a stable calibration quality for at least 20 weeks. Di Ninni et al.^17^ also observed high stability for the optical properties of Intralipid 20% (Fresenius Kabi) sample-to-sample and between batches and over time. Multiple samples were taken from the same batch for a 2-year period. A batch-to-batch variation of 2% was found for the reduced scattering coefficient and overtime only a difference of 4%. No significant differences were found in the sample-to-sample measurements.

In contrast, the clinically performed calibrations were not performed under uniform conditions. Therefore, the effect of the intralipid phantom could not be evaluated. However, it does provide the opportunity to assess the influence of the system, probe performance, and user performance (including tripod) over a prolonged period of time. We expected that probe degradation would influence calibration quality due to the induced stress, while the fiber tree was expected to be a passive component, not affecting the calibration. However, the probes did not show significant changes in performance over a period of time, but the fiber tree did. This illustrates that the fiber tree should be included in the quality assessment of the system. Moreover, the induced stress and usage of more than a hundred times did not affect the performance of the clinical probes. Although the probe performance is stable over time, we do not know if there is a first use effect. Since probes are manufactured and are sterile probes, it could be that their calibration properties change after their first contact with liquid. This first use effect could explain the high variability that was measured in the unused clinical probe. Therefore, an initial measurement of both the probe and fiber tree performances would be an important component of the quality assessment. This initial measurement could enable the calculation of a correction factor, which can be used to compare between different system configurations or probes. Finally, no difference in calibration quality was observed between users, and also the tripod did not show a quality improvement. This suggests that users can accomplish a stable measurement without the use of the tripod. However, this does not indicate that the tripod is not useful. While it may not aid in calibration stability, it does help users to make the calibration procedure easier to perform alone and allows them to perform other tasks during the calibration procedure.

The effect of the calibration quality on the optical properties could only be determined for the phantom experiment as these were performed under uniform circumstances. Intralipid phantom 8, which showed a lower SNR, caused deviations in the determination of the scattering properties. The decrease in SNR resulted in a large increase in the measured reduced scattering coefficient and a decrease in the power law exponent parameter for the skin. The magnitude of the deviation was different per probe. The details of how a contaminated 2% Intralipid phantom affects its scattering properties is unknown.

Translation of the effect of the calibration quality on clinically measured optical properties is challenging. Whereas the skin measurements were performed under uniform conditions, on the same tissue, and by one user only, clinical measurements are not. They are performed on various tissue types by multiple users and the measurements are sensitive to the movement of both the probe and the tissue underneath. Therefore, deviations in the optical properties are not only the result of the calibration but a combination of the calibration and the above-mentioned influences. Brooks et al. investigated the variability in the tissue optical properties of skin measurements performed by multiple users using MDSFR/SFF.^22^ This study focused only on probe placement and movement, pressure, and user variability, but not in the calibration itself. Variation of the measured optical properties was primarily found to be the result of tissue heterogeneity instead of user variability. In the present study, user variability was mainly defined as the variability in probe placement technique. Since Brooks et al could not prove that probe pressure caused the differences between users, they stated tissue heterogeneity to be the cause. It would have been interesting to confirm the calibration quality was the same for all measurements.

It is important to note that the minimum optical property value that can be measured is dependent on the SNR of the in vivo reflectance measurement. The reflectance SNR is affected by the accuracy of the calibration, fiber diameter, tissue type, and the integration time of both the calibration and measurement. For example, a poor calibration will have the strongest relative effect on $\mu_{s}^{\prime }$ followed by the power law exponent. The effect on the $\mu_{a}$, BVF, and ${\mathrm{StO}}_{2}$ will be less significant. As such, in the present study the variation we see in the scattering properties of skin probably overestimates the magnitude of the effect on $\mu_{a}$, BVF, and ${\mathrm{StO}}_{2}$. Our previously performed pilot studies have, to date, including more vascularized tissues (i.e., rectum, esophagus, and head and neck). In these studies, differences in $\mu_{a}$ have a larger influence on the correction for intrinsic fluorescence than differences in $\mu_{s}^{\prime }$.^18^ So, the effect of lower quality calibrations in the present study overestimates the magnitude of the effects of calibration quality in more highly vascularized tissues. However, in studies where the scattering properties are applied as a discriminator between tissues, like pancreatic tissue and head and neck lymph nodes,^2^^,^^23^ the effect of the calibration quality could be more important. We note reproducible data are relatively simple to measure in the normal skin,^22^ it might prove more challenging to acquire data from tissues with a higher blood volume to verify the mentioned influences. The oral cavity may be a potential area to perform future research. Additionally, in clinical use, the measurement integration time is the main limiting factor for increasing the SNR. The clinicians are limited by its ability to hold the probe stationary during the measurement. The integration time is therefore a trade-off between the longest possible integration time and the ability to keep the probe stable. An increase in the integration time would lead to a higher SNR and therefore allows a more accurate measurement of low BVF and StO_2_.

Our current proof-of-principle clinical studies are often short-term studies that include a small number of users and clinical subjects that normally utilize one imaging system.^4^^–^^8^ Therefore, the influence of the calibration quality was not our main focus. However, the potential for multicenter studies increases the need for comparison between systems and their functioning over time. The previously described results show that quality control/assessment will be important to give users feedback on the system performance and calibration quality over a longer period of time. For the current calibration procedure, the measured water spectrum can already indicate probe breakage by high peaks in the normally flat spectrum. However, it would be advantageous to know if degradation of the probe and/or fiber tree occurs before it breaks. Optimally, the calibration quality is calculated after each calibration and shown together with the initial calibration and previously performed calibrations. Further research on this data could maybe predict a cut-off value until when a probe or fiber tree can be used before it needs changing. Tracking the probe performance more carefully could also help the user with the interpretation of the clinical data. For example, if data deviates or lower values are measured than expected, the quality control can be assessed to see if there were any performance problems on the day of measurement. In addition to maintaining the performance of the device, the sterility of the intralipid phantom is also important. Even though the intralipid phantoms showed to be very stable over a long period of time, a contaminated phantom could have a large influence on the calibration quality. Especially in larger studies when both in vivo and ex vivo fibers are calibrated, it is important to regularly change the intralipid solution to prevent contamination. The frequency should rather be based on the number of calibrations performed with the phantom than the duration of storage, as the number of calibrations is a higher risk for contamination.

## 5 Conclusion

This study investigated which internal and external factors influence the calibration quality and optical property determination using the MDSFR/SFF spectroscopy system. The intralipid phantom quality and the fiber tree performance were the main factors influencing the calibration quality. Probe and user performance did not show any effect, which makes the upscaling of research to multicenter trials easier. Furthermore, a high-quality assessment procedure should be implemented to track changes during clinical trials over a longer period of time.
